# Fungi and Stone Heritage Conservation: Friend, Foe, or a Bit of Both

**DOI:** 10.3390/jof12020128

**Published:** 2026-02-11

**Authors:** Diana S. Paiva, Luís Fernandes, António Portugal

**Affiliations:** 1 Centre for Functional Ecology (CFE)—Science for People & the Planet, Department of Life Sciences, University of Coimbra, Calçada Martim de Freitas, 3000-456 Coimbra, Portugal; 2 TERRA—Associate Laboratory for Sustainable Land Use and Ecosystem Services, Department of Life Sciences, University of Coimbra, Calçada Martim de Freitas, 3000-456 Coimbra, Portugal

**Keywords:** biodeterioration, bioprotection, cultural heritage, fungi, stone conservation

## Abstract

The presence of lithobionts has historically been associated with biodeterioration, posing significant challenges to the conservation of culturally and historically significant stone heritage. This perception stems from abundant evidence of their role in biogeophysical processes, such as mechanical disruption of stone structures, and biogeochemical processes, which chemically alter stone composition through metabolic activity. These processes, while integral to natural systems, often accelerate the weathering and deterioration of heritage materials. Coupled with the aesthetic impact of lithobiont growth, frequently resulting in discoloration or obscuring of intricate details, such effects have justified the widespread removal of these organisms from heritage surfaces. However, recent research has revealed a far more nuanced picture. These communities can enhance biodiversity, contribute to the perceived authenticity of aged monuments, and, in some cases, form a biological layer that shields stone from pollutants and weathering forces. Moreover, developments in biomediated conservation approaches, such as biocementation and biocleaning, highlight their potential as sustainable allies in preservation. This dual role of lithobionts—both as friends and foes in preservation—is central to this review. This review focuses on how these organisms—with a particular emphasis on fungi, often perceived as enemies of conservation—may also serve as unexpected partners in safeguarding our stone heritage, emphasizing the need for case-by-case evaluation of active communities and their environmental context.

## 1. Introduction

Stone monuments and sculptures, cherished as cultural treasures, are both witnesses to history and canvases for nature’s unrelenting forces. While stone is often celebrated for its resilience and timeless appeal, it is far from impervious to the effects of time and environmental exposure. Microorganisms, including fungi, have long been recognized as key agents of stone biodeterioration, a role widely recognized and thoroughly documented in numerous studies [1,2,3,4,5,6,7,8,9,10,11]. Yet, in recent years, a growing body of research has highlighted their potential role as allies in stone conservation, sparking interest in concepts such as bioprotection, biological shielding, biocleaning, and bioconsolidation.

Fungi are often perceived negatively by the general public, frequently associated with decay, disease, or contamination. Still, they are essential to Earth’s fundamental cycles, driving geological transformations and nutrient recycling through the decomposition and reprocessing of organic and inorganic matter. Beyond their ecological significance, fungi have profoundly benefited humanity, contributing to the creation of foods and beverages like bread, cheese, and beer, as well as pioneering medicines such as antibiotics. The duality of these remarkable organisms has long been recognized in many fields, offering both challenges and significant advantages—so why not in the context of biodeterioration vs. bioprotection of stone cultural heritage?

Shielding (or umbrella-like protective effect) refers to the ability of microbial communities to form a protective layer, sheltering stone surfaces from further deterioration by external agents [12,13]. Biocleaning, on the other hand, involves the deliberate use of microorganisms to remove harmful substances, such as salts or pollutants, from stone materials [14,15,16]. Finally, bioconsolidation aims to enhance the structural integrity of deteriorated stone through microbial activity, typically by inducing the precipitation of mineral compounds within the stone matrix [17,18].

While biodeterioration has been extensively studied and documented, the dual role of microorganisms—both as culprits and sources of solutions—remains underexplored. This mini review delves into the emerging understanding of fungi and other microbes as agents of bioprotection, emphasizing their possible contributions to sustainable and environmentally friendly conservation strategies. By shedding light on these processes, we aim to demonstrate how the interplay between biology and stone conservation could offer innovative solutions to preserving cultural heritage for future generations.

## 2. Biological Colonization and Its Impact on Stone Decay

The colonization of stone heritage by living organisms is a natural process that occurs when the substrate is bioreceptive and environmental conditions are conducive to growth. Several factors influence the ability of stone surfaces to support living communities, including mineral composition, nutrient availability, pH, salinity, surface texture, moisture levels, porosity, permeability, and both climatic and micro-environmental conditions [19]. The stone’s mineralogical characteristics, surface properties, and environmental context collectively determine its bioreceptivity (its ability to be colonized), while the extent of colonization is shaped by environmental factors such as pollutant concentrations, microclimatic conditions, and anthropogenic atmospheric eutrophication [6,20]. Biological colonization, bioweathering, and biodeterioration in outdoor environments are largely driven by climate and location, whereas in indoor settings, they are primarily influenced by human occupancy and associated activities [21].

Conventionally, the presence of organisms on monumental stones and artworks has been directly associated with deterioration processes [1,19,22,23]. However, their presence does not inherently imply that biodeterioration is driven by their activity. Recent studies have sparked debates about the potential protective effects of some organisms against environmental deterioration [24,25,26]. It seems that the two are not mutually exclusive and that whether biodeterioration or bioprotection occurs depends on the climatic conditions and the characteristics of the substrate, as well as on the types of organisms present [12,27].

The alteration of stone substrates is a complex process driven by abiotic factors and biotic processes, which frequently act synergistically. This dynamic interplay makes it challenging to evaluate the relative contribution of biological and non-biological factors in the deterioration processes [2]. In natural environments, the physico-chemical alterations induced by living organisms—such as the transformation of rocks into soil—fulfill an essential and beneficial ecological function. Conversely, when such interactions occur on heritage stones, the relationship between the colonizing organisms and the substrate can become detrimental.

**Figure 1:**
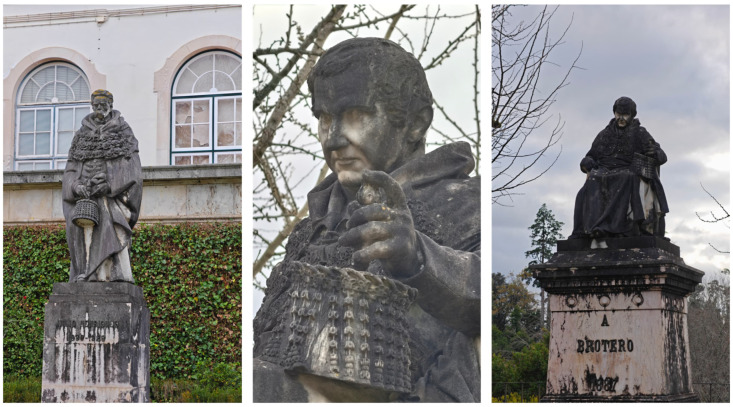
Examples of monumental stone statues exhibiting biological colonization.

Stone heritage is colonized by a diverse range of organisms, including macroorganisms such as vascular plants, bryophytes, and lichens, as well as microorganisms such as fungi, microalgae, cyanobacteria, and bacteria, which are often organized into biofilms. These organisms interact with inorganic substrates in various ways, leading to discoloration, soiling and encrustation, salting and mineralization, mechanical damage including disruption or distortion, cracking and fragmentation, acid attack and interactions with ions (Figure 1) [6,28,29,30,31,32].

## 3. The Role of Fungi in Stone Biodeterioration

Among the various organisms mentioned above that can colonize stone and contribute to its deterioration, this work will primarily focus on fungi, though it is important to acknowledge the role of other colonizers and their combined effects in the context of biodeterioration and bioprotection.

Fungi represent an extraordinarily diverse group of eukaryotes, capable of thriving in virtually every ecological niche [33]. Although stone is regarded as a challenging habitat—characterized by nutrient scarcity, fluctuating moisture levels, and many other adverse conditions—fungi have been documented colonizing stone surfaces across all climatic regions worldwide [34,35,36]. They thrive on both natural and man-made stone materials, in both indoor and outdoor environments, and are widely recognized as one of the most important and impactful stone colonizers. Their activity plays a pivotal role in the biodeterioration of stone substrates, including culturally significant heritage artifacts [30,37]. As chemoheterotrophic organisms, their metabolic adaptability surpasses that of many other biodeteriogens, allowing them to colonize a broad range of organic and inorganic substrates [38]. This versatility, coupled with their ability to withstand extreme environmental conditions, form mutualistic relationships, and adopt diverse structural, morphological, and metabolic strategies, makes them exceptionally skilled at colonizing stone materials [7,21,38]. As heterotrophic organisms, fungi cannot directly metabolize inorganic substrates. However, they can thrive on organic residues, such as discarded products and/or decayed organic matter of previous/additional communities or other sources, bird droppings, and aerosols from other organisms also found on stone surfaces, as well as alternative carbon sources like rainwater, groundwater, and fossil organic carbon embedded in biogenic rocks [5,7,21,37,39,40].

Stone-colonizing fungi can be categorized based on their ecology and taxonomy. Ecologically, they are divided into epilithic fungi, which grow on stone surfaces, and endolithic fungi, which inhabit internal pores and fissures. Endolithic fungi are further subdivided into chasmoendoliths, cryptoendoliths, and euendoliths, depending on their specific mode of colonization within the stone. Taxonomically, fungal communities on stone monuments can be grouped into two main categories, each adapted to different environmental conditions. In humid, temperate climates, filamentous fungi such as Hyphomycetes and Coelomycetes predominate, whereas arid and semi-arid regions are typically dominated by black microcolonial fungi (MCF) and yeasts [5,35]. These ecological and taxonomic differences arise from the varying nutrient requirements and adaptive traits of these groups. Hyphomycetes and Coelomycetes thrive in nutrient-rich conditions, while MCF and yeasts have unique physiological and morphological adaptations that allow them to survive in harsh, nutrient-poor environments [7,35]. Species of *Alternaria*, *Cladosporium*, *Epicoccum*, *Aureobasidium*, and *Phoma* are frequently found among the most common Hyphomycetes and Coelomycetes, while black fungi from genera such as *Hortaea*, *Sarcinomyces*, *Coniosporium*, *Capnobotryella*, *Exophiala*, and *Trimmatostroma* form compact black colonies both on and within stone surfaces and often occur in close association with lichens [5].

Coelomycetes and both hyaline and dematiaceous Hyphomycetes are commonly isolated from cultural heritage materials globally [41]. Their biodeteriorative activities primarily involve physical damage caused by hyphal penetration into the substrate and the production of extracellular corrosive metabolites [19,41,42,43]. Additionally, their high pigment production results in visible discolorations, such as black spots and colored patinas, significantly affecting the aesthetic integrity of stone [5,37]. Some species further contribute to chemical damage through the production of organic acids and enzymes [30,44,45]. Over extended periods of time, these fungi influence microbial community dynamics, supporting biofilm development through the secretion of extracellular polymeric substances (EPS) [29].

On the other hand, MCF, also referred to as microcolonial fungi, black yeasts, meristematic fungi, or rock-inhabiting fungi (RIF), represent a phylogenetically diverse and ecologically convergent group adapted to extreme and hostile environments [35,36,46]. These fungi are primarily classified within the classes Dothideomycetes (orders Capnodiales, Dothideales and Pleosporales) and Eurotiomycetes (order Chaetothyriales) [47]. Their distinctive morphophysiological traits include highly melanized cell walls, slow growth, morphological plasticity, the ability to transition between mycelial and meristematic states, simple life cycles, and dispersal through vegetative fragmentation or poorly differentiated conidia-like cells [35,36,47,48]. These features allow them to thrive under harsh conditions, such as nutrient scarcity, intense UV radiation, temperature fluctuations, osmotic stress, and severe desiccation [36,49]. The prevalence of melanin in their cell walls is a hallmark of MCF, providing critical stress protection while also conferring mechanical strength to the hyphae. Alongside melanin, other protective compounds such as mycosporines and carotenoids also contribute to their survival under unfavorable conditions [35]. As a result, MCF successfully colonize extreme habitats like deserts, saltpans, contaminated sites, and stone surfaces, where other fungi struggle to survive [50]. Their remarkable resistance has earned them recognition as some of the most resilient eukaryotic organisms known so far [51].

Beyond their survival skills, MCF play an active role in stone biodeterioration. They produce extracellular polysaccharides that corrode surfaces, create microcavities, and deepen fissures, allowing penetration into the rock matrix, where they find some degree of protection [35,52,53,54]. Melanin, in addition to causing visible aesthetic alterations, provides additional mechanical strength to the hyphae, allowing them to penetrate further into the fissures and intercrystalline spaces, which facilitates crystal detachment and the formation of surface micro holes [55]. While melanization of fungal cell walls was once thought to be critical for mechanical penetration, recent research on *Knufia petricola* A95 by Tonon et al. [56] highlighted that hyphal morphology and substrate porosity, rather than melanization alone, are more critical factors in this process.

Given their extreme resilience and destructive potential, MCF are considered one of the most challenging groups to manage in the preservation of stone cultural heritage, posing a significant threat to monuments and artifacts worldwide [55,57,58].

Fungal-mediated deterioration of stone generally combines aesthetic alterations with biophysical and biochemical processes, as briefly mentioned earlier.

### 3.1. Aesthetic Deterioration

Surface colonization by fungi and other organisms often results in some of the most apparent and initially observed damage, particularly aesthetic alterations. These changes are primarily driven by color shifts caused by the presence of melanin and other pigments in fungal cells, their contribution to biofilm formation, and their interactions with inorganic compounds [5,6,30]. In biofilms, pigmentation and coloration are influenced by both the organisms present and their physiological state, as well as by environmental factors such as radiation type and intensity, temperature, and humidity. These combined effects result in stone discoloration, which is frequently regarded as visually undesirable.

### 3.2. Biophysical Deterioration

Biophysical deterioration refers to the mechanical stresses caused by living organisms that lead to the mechanical fracturing of stone and minerals [59]. This phenomenon results from their settlement and development, which induce fractures in the substrate either through the development of structures such as hyphae and/or volumetric changes, as observed in biofilms. Several fungal species actively penetrate the stone matrix in search of nutrients, physically compromising its structural integrity [60]. Additionally, the contraction and expansion of hyphae under fluctuating environmental conditions apply mechanical pressure, further damaging the stone matrix [5,37]. Biofilms also exert stress on the substrate through the swelling and contraction of their EPS, particularly during cycles of hydration and desiccation or freezing and thawing. When such processes occur within microcracks, they can exacerbate fissures and alter water flow patterns within the substrate [61,62]. This biophysical weathering increases the exposure of minerals within the stone to organisms and deteriogenic agents, thereby facilitating subsequent deterioration processes.

### 3.3. Biochemical Deterioration

Biochemical deterioration, as described by Silverman [59], encompasses processes driven by biological colonization that alter the chemical composition of stones and their constituent minerals. These processes are closely tied to the metabolic activities of organisms as they acquire energy and essential chemical elements for growth.

Fungi (including lichenized forms, though not the focus here) exhibit a remarkable ability to induce biochemical deterioration through the excretion of various compounds, including extracellular mucilaginous substances, organic acids and chelating agents, favoring mineral dissolution, surface pitting, displacement, precipitation and neoformation of salts, as well as the oxidation of cations, thereby contributing to the deterioration of mineral substrates [5,7,21,37,39,42]. Among these mechanisms, the most significant impact is often attributed to their secretion of a wide range of carboxylic acids, including citric, succinic, formic, malic, acetic, fumaric, glyoxylic, gluconic, tartaric, and especially oxalic acid, which acidify their immediate environment. Upon contact with the substrate, these acids initiate a chemical attack that dissolves minerals and promotes the formation of secondary minerals, while simultaneously releasing essential nutrients that sustain fungal growth and metabolism [5,30,37,43,63,64,65]. Interestingly, recent research by Li et al. [66] on *Talaromyces flavus* revealed that the production of organic acids is highly mineral-specific, with the type of substrate influencing the fungal response. This finding highlights the adaptability of fungi to different environmental conditions and their tailored mechanisms for substrate degradation.

In addition to organic acids, fungi produce other metabolites with chelating properties, such as amino acids, siderophores, and phenolic compounds, although their mechanisms of action are less well characterized. Siderophores, for instance, exhibit high specificity for chelating or binding iron-related ions [7,21]. These compounds are typically synthesized in environments with limited iron availability, enabling fungi to scavenge this essential element and render it accessible for their metabolic needs [67]. Favero-Longo et al. [53] demonstrated that certain microcolonial fungi, such as *Knufia petricola*, can penetrate stone surfaces through the production of siderophores.

Moreover, fungi can passively (metabolism-independently) associate metal elements in their cell walls via mechanisms like ion exchange, adsorption, complexation, precipitation, and crystallization, a process collectively referred to as biosorption. This interaction can further contribute to changes in stone structure, potentially weakening the substrate and further exacerbating deterioration [39].

Furthermore, biofilms, comprising both phototrophic and heterotrophic organisms, produce EPS, as noted earlier. These substances, primarily composed of water, prolong the water–substrate interaction [32], promoting chemical weathering processes such as hydration and hydrolysis.

### 3.4. Fungal-Induced Mineralization

Fungi not only contribute to the dissolution of minerals but also play a significant role in the formation of secondary minerals through a combination of biochemical and biomechanical processes, often referred to as fungal stone diagenesis. On carbonate substrates, this involves the precipitation of carbonates and/or oxalates during fungal colonization. The process begins with the dissolution of the carbonate substrate, primarily driven by organic acids secreted by fungi, and is followed by the reprecipitation of new minerals through secondary biomineralization occurring around fungal hyphae at the interface with the stone matrix [7,21,39,68]. This biomineralization relies on the ability of fungi to form complexes with various metals, including calcium, magnesium, manganese, zinc, copper, aluminum, and iron. The type of secondary minerals formed depends on the mineralogical composition of the substrate, but they are commonly calcium and/or magnesium oxalates, such as weddellite and whewellite, and carbonates, such as calcite [7,21].

Over time, the precipitation and accumulation of these minerals can help fill and stabilize cracks and fissures in the substrate, effectively acting as a cementing mechanism [63].

## 4. Fungi: A Double-Edged Role

The presence of lithobionts (organisms living on stone surfaces) has historically been associated with biodeterioration, posing significant challenges to the conservation of culturally and historically significant stone heritage [69]. This perception stems from abundant evidence of their role in biogeophysical processes, such as mechanical disruption of stone structures, and biogeochemical processes, which chemically alter stone composition through metabolic activity [59,70]. These processes, while integral in natural systems, often accelerate the weathering and deterioration of heritage materials. Coupled with the aesthetic impact of lithobiont growth, frequently resulting in discoloration or obscuring of intricate details, such effects have justified the widespread removal of these organisms from heritage surfaces [71].

However, the role of lithobionts in stone heritage conservation is increasingly recognized as more complex than initially believed. While their deteriorative potential is well documented, studies have also highlighted their potential for bioprotection. This ambivalent role has been hypothesized since the late 20th century [25,72], with some research demonstrating bioprotective effects under certain conditions. Lithobiontic communities, for instance, may contribute to biodiversity and, in some cases, enhance the perceived aesthetic value of cultural heritage, providing a visual sense of age and historical authenticity. A monument weathered over centuries, after all, should not appear as though it were constructed yesterday. Recent studies have further emphasized the protective potential of these communities. They can form an umbrella-like layer that shields stone surfaces from abiotic weathering agents, such as atmospheric pollutants and meteorological forces, acting as a natural barrier against environmental degradation [12,13]. Additionally, advances in biomediated approaches have sparked interest in using microorganisms to consolidate stone (e.g., biocementation) or even to clean heritage surfaces through specific metabolic activities (e.g., biocleaning; [14,17,18]). Such findings suggest that, under appropriate conditions, bioprotection could represent a sustainable conservation strategy [24].

This dual role of lithobionts—both as friends and foes in preservation—will be further explored in this review. The analysis will focus on how these organisms (with particular emphasis on fungi), often perceived as enemies of conservation, may also serve as unexpected partners in safeguarding our stone heritage, emphasizing the need for a case-by-case evaluation of the active communities and the specific microenvironments or climatic conditions surrounding these historical assets.

## 5. Bioprotection—A Multidimensional Approach to Heritage Conservation

Bioprotection, an Earth surface process, encompasses the protective effects mediated by the growth of living organisms, their remains, or their metabolic byproducts [12]. While originally associated with natural rock surfaces, the concept has been adapted to the built environment, including cultural heritage assets [14,24,25]. Within this context, bioprotection may involve cleaning, consolidation, or the “shielding/umbrella” effect on stone substrates from abiotic weathering and biodeterioration.

Efforts to prevent, control, or remediate microbial colonization and its deleterious effects on stone heritage have employed diverse strategies, yet fungi often present significant challenges [73]. Preventative measures typically aim to inhibit biological growth by altering environmental conditions such as nutrient and moisture availability, while control approaches target the eradication of colonizers, restoration of the artifact, and suppression of recolonization [71]. These interventions generally focus on disrupting microbial vitality and functionality, coupled with measures to slow reestablishment. Conservation strategies, spanning cleaning, disinfection, restoration, and protection, must be tailored to the specific characteristics of the heritage material. These efforts must be complemented by preventative maintenance practices to improve their effectiveness. Additionally, monitoring the long-term effects of treatments is essential to ensuring their sustainability and success [38].

Preventive approaches must be adapted to the specific origin and location of the material, as environmental factors significantly influence its preservation. For indoor environments, effective prevention can include regular dust removal, stabilization of climate conditions, adequate ventilation, regulation of light exposure, and implementing specific restrictions for visitors and staff [71]. Conversely, outdoor environments present greater challenges due to the multitude of factors that promote microbial colonization and growth. Simple measures, such as controlling animal activity, reducing moisture on stone surfaces, repairing drainage systems, and clearing excessive soil and debris, can effectively limit further biodeterioration [74,75]. In addition, more complex interventions can be employed, including the use of insulation materials, constructing protective shelters, and applying water repellents and nanoparticles, among others [71,75,76,77,78,79].

In turn, control methods encompass physical, chemical, and biological approaches [1,14,73,80,81,82,83]. Physical procedures can include the use of electromagnetic wavelengths such as ultraviolet light and LEDs, laser cleaning, and gamma radiation [71,84,85], as well as temperature-based techniques like heat shocking, microwaves, and dry ice treatments [86]. Mechanical cleaning methods, involving brushing, sandblasting, water-based techniques, and vacuuming, are also commonly employed [71,87,88,89,90]. While these approaches avoid risks to human health and the environment, they often present significant limitations. Their aggressive nature can lead to severe structural and aesthetic damage, such as the loss of carved details, and they are frequently inadequate for addressing endolithic microorganisms that reside deep within materials [88,89,90].

Biocides are the most commonly employed method for controlling biodeterioration in cultural heritage contexts. While their effectiveness is undeniable, their use carries significant challenges, including risks to visitors, staff, the environment, and the integrity of the stone itself [71,91,92,93,94,95]. Additionally, frequent reapplications are often necessary to prevent microbial recolonization, which can amplify their negative impacts. Improper or poorly managed application of biocides can have catastrophic consequences, such as inadvertently promoting the survival and proliferation of resistant microbial strains that accelerate biodeterioration. Striking examples of such unintended consequences are the infamous cases of the Lascaux Caves in France and the Cave of St. Paul in Turkey [38,73,96,97], where chemical interventions not only failed to control microbial growth but also resulted in both structural and aesthetic damage due to the emergence of more aggressive biodeteriorative agents. These incidents highlight the potential dangers of chemical treatments, underscoring the need for careful evaluation and application. To mitigate such risks, an ideal biocide must effectively target harmful microorganisms while ensuring safety for visitors and staff. It should also have prolonged efficacy, minimize environmental impact, and avoid causing any damage to the stone or inadvertently enhancing microbial recolonization [75,87,98].

In contrast, biological methods leveraging microorganisms and enzymes for biorestoration emerge as viable alternatives. These approaches mimic natural processes under controlled conditions and offer several advantages, including minimal invasiveness, reduced health risks for conservators, and environmental safety [73,99]. By avoiding the harsh impacts of mechanical and chemical methods, biological interventions provide a softer, eco-friendly solution to conservation challenges, fostering sustainable preservation of cultural heritage.

### 5.1. The Umbrella Effect

Although the involvement of lithobionts in the weathering of natural and artificial stones is well documented, growing evidence also points to their bioprotective roles (an idea hypothesized decades ago by Krumbein [72]). Thus, the axiomatic association between these colonizers and stone weathering remains a matter of controversy.

Microbial communities present on stone surfaces represent complex, multispecies ecosystems, with colonization occurring under favorable conditions (e.g., adequate moisture, light, temperature, and nutrient availability). When present, understanding their effects requires a comprehensive assessment of the extent to which they contribute to material deterioration, as well as an assessment of the non-biogenic agents that also take part in the deterioration process and their simultaneous interactions [100]. Many biotic and abiotic factors have similar effects, acting in synergy or independently in quantitatively variable relations. Consequently, evaluating the overall impact of microorganisms on stone deterioration requires careful consideration, as their mere presence does not necessarily imply significant changes to the material’s physical or chemical properties [27]. Natural weathering processes (such as erosion, decay, and material breakdown driven by abiotic factors) coexist with microbial activity, creating a dynamic and unstable equilibrium. This balance can shift depending on environmental conditions, substrate characteristics, and the specific organisms colonizing the surface [101].

The hypothesis of lithobionts providing bioprotection emerged from early observations of differential erosion rates between colonized and uncolonized surfaces, suggesting an “umbrella effect” that shields stones from external weathering agents [102,103]. Evidence supporting this protective effect includes studies like McIlroy de la Rosa et al. [13], which demonstrated lower solutional weathering on lichen-covered limestone slates compared to uncolonized controls after a year of exposure in a humid Irish climate.

Lichens and biofilms are the colonizers that have received the most attention in this regard [73]. For example, non-colonized stone surfaces often exhibit increased rates of exfoliation, flaking, and saline efflorescence compared to areas colonized by lichens [27]. Lichens can reduce wind and rain impact [104,105] and limit erosion by reducing the level of water within the stone [106]. Carter and Viles [107] observed that moisture retention within the lichen thallus helped mitigate thermal stress on a limestone surface. Studies by Ariño et al. [101] and Wendler and Prasartet [108] suggest that lichens may slow the decay of porous stones by reducing water exchange between the stone and the surrounding environment or by buffering damage from atmospheric agents like pollutants. Moreover, fungal- or lichen-induced biomineralization of secondary mineral precipitates (e.g., carbonates and oxalates) could serve as both consolidants and protective coatings, further stabilizing and protecting stone surfaces [24,109]. In arid climates, microorganisms colonizing rock surfaces have been shown to be chemically involved in the formation of rock coatings, which enhance surface stability and support long-term preservation [110,111]. The ability of melanins and EPS to bind metals, particularly around the cell walls of melanized fungi (MCF), has been linked to varnish formation. This process is further supported by observations of manganese oxidation and the presence of manganese-coated fungal hyphae, identified in cultures derived from rock varnish grown on manganese-enriched media [112]. Biofilms comprising cyanobacteria and fungi have also been found to contribute to the hardening of sandstone in arid regions such as Jordan by promoting cementation [113]. Similarly, in the cold and arid environment of central Antarctica, endolithic lichens and their EPS facilitate the biomineralization of iron oxides [114]. Interestingly, bioprotective effects extend to wetter climates as well. In the UK, epilithic lichens have been observed to deposit silica-rich layers within granite cracks and along mineral boundaries, reducing abiotic weathering impacts [115]. Additionally, the biomineralization of oxalates, such as calcium oxalates, has been proposed as a mechanism for forming protective shields against abiotic weathering agents like wind and water runoff [116,117].

However, the outcome of this bioprotection varies depending on substrate properties and environmental conditions, emphasizing the complexity of interactions between biotic and abiotic factors. For example, fungal excretion of organic acids can dissolve minerals but may also lead to biomineralization, such as the formation of oxalates or secondary calcite; these secondary minerals can act as protective crusts but may also damage the substrate through crystal expansion [116,118]. Furthermore, biodeteriorative actions likely persist, albeit at a slower pace compared to those driven by abiotic agents.

This simultaneous dual effect has been documented in several cases. For example, the lichen *Verrucaria rubrocincta* on caliche plates in the Sonoran Desert demonstrates concurrent substrate deterioration and biomineralization of a protective micrite layer [105,119]. Similarly, non-lichenized fungi have been shown to dissolve calcite while simultaneously promoting the formation of secondary calcite around their hyphae [63]. Cases like these, where studies recognize both protective and deteriorative effects simultaneously, highlight the need for detailed, site-specific investigations to evaluate the dominant processes. They also underscore the importance of understanding the spatial distribution of these effects, their persistence over time, and their actual impact on stone durability before making informed management decisions [27,118].

**Figure 2:**
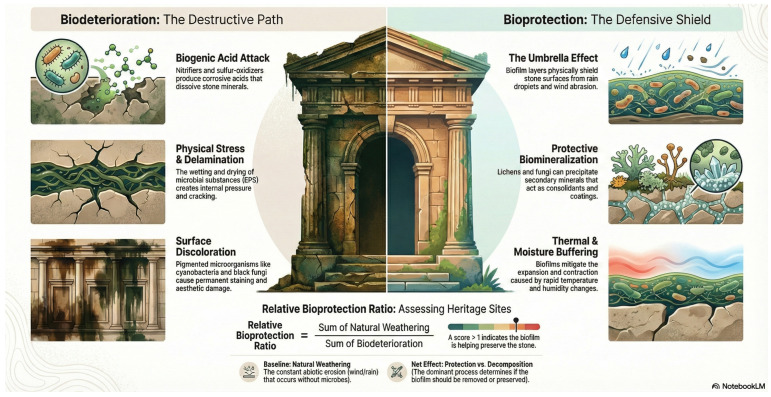
Conceptual representation of biodeteriorative versus bioprotective effects, adapted from the study by Liu et al. [26].

As discussed above, a variety of mechanisms may contribute to this complex interplay, making generalized predictions challenging. Liu and colleagues [26] proposed a “relative bioprotection ratio” to differentiate bioprotection from biodeterioration and abiotic weathering. This ratio compares the combined effects of natural weathering and biodeterioration, with values greater than one indicating bioprotection and values below one suggesting that biodeterioration is predominant (Figure 2). However, this definition relies on the ability to disentangle natural weathering from microbe-mediated activities and generate quantitative data—a challenging and often unattainable task in many contexts.

### 5.2. Biocleaning

Biocleaning utilizes the metabolic activities of microorganisms to remove inorganic and organic surface deposits, offering a sustainable method to clean or ameliorate affected surfaces [15]. This innovative approach has been applied to heritage materials, utilizing microbial systems to effectively eliminate pollutants and residues without compromising the integrity of the substrate [95,120,121]. Both microorganisms and their extracted enzymes have demonstrated success across various materials, including stone, paintings, paper, ceramics, concrete, statues, and frescoes, often outperforming traditional chemical and physical methods in terms of efficiency and gentleness [14,16].

Cleaning is a critical step in the restoration process and typically precedes other conservation interventions. However, for structures with delicate or intricate features, conventional physical and chemical cleaning methods can pose risks to the materials and the health of restorers [99]. Biocleaning offers a safer alternative, particularly for stone heritage, where bacterial systems, such as *Desulfovibrio* and *Pseudomonas* species [122], have been effective in removing nitrates and sulfates without adverse effects on materials or personnel [73,99]. Successful applications of biological cleaning techniques have been documented in cultural heritage sites across Italy, Spain, and Greece [123].

An alternative method, “dry biocleaning,” uses dehydrated *Saccharomyces cerevisiae* yeast applied to stone surfaces. Upon rehydration, the yeast’s metabolic activity effectively removes salts and pollutants, achieving better results compared to cell-free controls [95]. Beyond stone, biocleaning has shown promise for other materials. For example, alkaliphilic fungi have been employed to clean corroded iron surfaces, successfully removing powdery chlorinated corrosion layers without damaging the underlying metal [16,73]. These examples underscore the broader potential of biocleaning as an innovative, eco-friendly solution for heritage preservation.

### 5.3. Bioconsolidation

Biomineralization, the biologically mediated process of mineral formation, is a cornerstone of bioconsolidation techniques aimed at preserving cultural heritage structures. A consolidant, as defined by Wheeler [124], is a material or system that penetrates a substrate, improving its inner structure and mechanical properties while enhancing surface adhesion. Effective consolidants must satisfy key criteria, including compatibility with the original material, even penetration, durability, and retractability to ensure no adverse effects on prior treatments [125].

Bioconsolidation processes are grounded in biomineralization, which may play a dual role in the context of cultural heritage conservation. While certain biomineralization processes can contribute to the deterioration of stone surfaces (for example, via the oxidation or reduction of metallic elements constituent of the substrate), they can also lead to the formation of secondary mineral deposits. These precipitates, including carbonates, phosphates, oxides, and oxalates, can create robust varnish-like coatings or fill microcracks in the stone, thereby enhancing its structural integrity and coherence [26,29,43,63].

Bioconsolidation is achieved through the direct or indirect application of microorganisms and/or their metabolites [126]. A key process in bioconsolidation is microbially induced calcium carbonate precipitation (MICP), also known as biocalcification or biocarbonatogenesis. This eco-friendly method has proven effective for stabilizing decayed stones, particularly those composed of carbonate minerals such as limestone and marble, which are widely used in artworks and monuments [127]. By harnessing microorganisms’ ability to precipitate calcium carbonate, MICP mimics natural processes observed in environments like soils, natural rocks, caves, and aquatic systems [126]. This process facilitates the deposition of a coherent calcium carbonate layer, filling microcracks and enhancing the stone’s structural integrity while protecting it from water uptake. Ideally, it should result in a mineral layer that closely resembles the original stone substrate, a highly desirable result in the eyes of conservators [99].

MICP has been widely explored in bioprotection, with most research focusing on bacteria (such as *Bacillus* species, among others), while fungal systems remain less explored. Approaches include the direct application of carbonatogenic bacteria, enrichment of native microbial communities, and the use of cell-free bacterial products. In the construction industry, MICP has been applied to self-healing concrete for crack repair [128,129,130,131,132,133,134,135]. A common bacterial pathway involves microbial hydrolysis of urea, a process that increases alkalinity and promotes the precipitation of calcium or other metal carbonates [136]. This urease-mediated approach has been used to enhance the durability of structures by reducing water infiltration and corrosion [131,137], sealing cracks [138,139], and restoring historic monuments [140].

While the majority of bioconsolidation research has focused on bacteria, fungi have emerged as promising agents in recent years (e.g., *Penicillium chrysogenum* in Fang et al. [141], *Colletotrichum acutatum* in Li et al. [127], and *Paecilomyces inflatus* and *Plectosphaerella cucumerina* in Pasquale et al. [142]). Fungal systems offer advantages over bacterial counterparts, such as dense, fibrous mycelial networks and higher biomass, which enable deeper penetration and greater surface coverage, enhancing the mechanical strength and durability of treated substrates. Several fungal species exhibit high urease activity and tolerance to alkaline conditions, effectively mediating the precipitation of calcite and other metal carbonates [109]. For instance, *Neurospora crassa*, a urease-positive fungus, has demonstrated the ability to produce biomineralized coatings on porous materials like mortar and cement. Its hydrophobic mycelial network acts as a physical barrier to water infiltration, while calcium carbonate precipitated within pores and cracks serves as a biocement, reinforcing the substrate [143]. Additionally, fungi can precipitate stable secondary minerals such as calcium oxalates (e.g., whewellite and weddellite), which are often found in patinas on stone monuments that can also contribute to stone longevity [64,144]. Precipitation of secondary calcite after fungal dissolution of limestone can result in cementation of the original limestone substrate [145]. Moreover, several fungal species capable of oxidizing manganese (Mn) can also form Mn oxides, a key component of rock varnishes [29], further extending the potential applications of fungal biomineralization.

While fungal bioconsolidation remains underexplored compared to bacterial systems, its potential for addressing challenges in cultural heritage preservation is increasingly recognized. The ability of fungi to consolidate porous substrates and enhance resilience positions them as a valuable complement to bacterial approaches. Continued research into fungal biomineralization could lead to innovative, sustainable solutions aligned with the growing demand for environmentally friendly conservation methods.

### 5.4. Aesthetics in Perspective

Lithobiontic communities, although often associated with biodeterioration, also play a significant role in enhancing biodiversity (as evidenced in several studies [8,9,10,146,147,148]) and conceal reservoirs of still-undiscovered taxa [149,150,151,152,153,154,155,156,157,158,159,160,161,162,163]. In some cases, they may even enhance the perceived aesthetic value of cultural heritage, contributing to a visual sense of age and historical authenticity—a reminder that structures standing for centuries are not meant to appear as if they were built yesterday. As highlighted in the review by Favero-Longo and Viles [25] on the role and management of lithobionts in stone cultural heritage, informed decisions regarding their removal, preservation, or further encouragement require integrating aesthetic assessments with data on their impact on stone durability, carefully weighing their biodeteriorative and bioprotective roles. For example, if lithobionts are primarily bioprotective but cause significant aesthetic damage, their removal might compromise the structural durability of the stone. In such cases, strategies to replicate their bioprotective function should be implemented. Conversely, if their aesthetic impact is minor and evidence points to bioprotection or negligible effect, or only minor biodeterioration impact, these communities may well be preserved and their biodiversity promoted as an added value to the site. This approach is particularly relevant for cultural heritage stone structures in natural environments, where the removal of mature, climactic lithobiontic communities, resembling those on surrounding natural outcrops, often leads to rapid recolonization by less diverse and more opportunistic species [164], which can potentially have more detrimental effects. Including biodiversity in the cultural value of heritage sites aligns with conservation frameworks that integrate cultural and natural heritage. Such strategies ensure a balanced assessment of risks and benefits, enabling broader and more sustainable conservation efforts.

### 5.5. Biocontrol Agents

A distinct bioprotection application is the use of entomopathogenic fungi to control insect and other arthropod infestations (key vectors for the dispersion of many stone-colonizing species) in indoor environments, including museums, libraries, and archives. These fungi, widely used to reduce post-harvest insect and mite populations, offer an eco-friendly alternative to toxic chemicals, with products targeting larvae or adults of various pest species [165]. While generally tested for safety in humans, their application in environments containing organic materials, such as books and paintings, raises concern about their potential to act as saprophytes and degrade the materials they aim to protect. Pinzari et al. [166] studied a strain of *Metarhizium anisopliae*, a known biocontrol agent used in various formulations, to assess its suitability for cultural heritage applications. The strain was tested against paper-degrading insects from the family *Anobiidae* and evaluated for its potential to degrade paper. Phenotypic microarrays and enzyme activity tests revealed a metabolic profile compatible with safe use in libraries and archives. These results indicate potential avenues for exploring new fungal bioprotection strategies in the cultural heritage sector.

## 6. Charting New Directions in Stone Conservation

While significant strides have been made, the study of stone-colonizing microorganisms remains a field with untapped potential. Many organisms’ influence on stone heritage has yet to be identified, and the intricate mechanisms behind their interactions with stone surfaces demand further exploration.

While fungi have traditionally been viewed as culprits of damage, their dual role as potential protectors offers an exciting avenue for research and application. The integration of cutting-edge methodologies, alongside the development of standardized tools for assessing microbial impacts, can further refine our understanding of these complex processes. Future studies should aim to bridge the gap between laboratory insights and real-world applications, moving toward sustainable and adaptive conservation practices. To bridge these knowledge gaps, researchers advocate for the creation of globally accessible databases cataloging both the detrimental and beneficial roles of these colonizers.

By embracing the duality of microbial interactions with stone, we can work toward innovative solutions that not only mitigate biodeterioration but also harness the potential of microorganisms as allies in preserving our cultural heritage.

## Data Availability

No new data were created or analyzed in this study. Data sharing is not applicable to this article.

## Abbreviations

The following abbreviations are used in this manuscript:

LED	Light-emitting diode
EPS	Extracellular polymeric substances
MCF	Microcolonial Fungi
UK	United Kingdom
MICP	Microbially induced calcium carbonate precipitation
RIF	Rock-inhabiting fungi
